# Association between sleep pattern and pharmacological treatment in children with attention deficit disorder with hyperactivity: a systematic review

**DOI:** 10.1590/1984-0462/2023/41/2022065

**Published:** 2023-05-29

**Authors:** Nathalia Sena Rocha, Rogério do Espírito Santo Amorim Correa, Adria Carolina de Melo Dias, Cláudia Dizioli Franco Bueno

**Affiliations:** a Universidade do Estado do Pará, Marabá, PA, Brasil.

**Keywords:** Attention deficit disorder with hyperactivity, Drug therapy, Sleep, Transtorno do déficit de atenção com hiperatividade, Tratamento farmacológico, Sono

## Abstract

**Objective:**

The aim of this study was to analyze the effect of the pharmacological treatment on the sleep patterns of children with attention deficit hyperactivity disorder (ADHD).

**Data source:**

A high-sensitivity electronic search was performed in the following databases: Cochrane Library, MEDLINE via PubMed, LILACS via the Regional Health Portal (BVS), Embase, Scopus, CINAHL, and Web of Science, as recommended by the Cochrane Handbook, and which has undergone peer review according to the PRESS Guide.

**Data synthesis:**

The studies contemplated the use of the drugs atomoxetine, guanfacine, methylphenidate, dasotraline, L-theanine, and lisdexamfetamine. They showed efficiency in reducing the symptoms of ADHD, although all, except atomoxetine, affected sleep quality, such as by reducing total rapid eye movement (REM), non-REM phase, slow-wave sleep time, and longer sleep-onset latency.

**Conclusions:**

The drugs used in the treatment of ADHD seem to have negative repercussions on the sleep quality of children, with the drug atomoxetine showing lesser effects on this variable.

## RESUMO

**Objetivo:**

Analisar a interferência do tratamento farmacológico no padrão de sono das crianças com transtorno do déficit de atenção com hiperatividade (TDAH).

**Fontes de dados:**

Busca eletrônica de alta sensibilidade nas bases de dados Cochrane Library, Medical Literature Analysis and Retrieval System Online (MEDLINE) via United States National Library of Medicine (PubMed), Literatura Latino-Americana e do Caribe em Ciências da Saúde (LILACS) via Biblioteca Virtual em Saúde (BVS), Embase, Scopus, Cumulative Index to Nursing and Allied Health Literature (CINAHL) e Web of Science, de acordo com o preconizado pelo Cochrane Handbook, de artigos que passaram pela revisão por pares segundo o Guia PRESS.

**Síntese dos dados:**

Os estudos que contemplaram o uso das drogas atomoxetina (ATX), guanfacina, metilfenidato (MPH), dasotralina, L-teanina e lisdexanfetamina mostraram eficiência na redução dos sintomas do TDAH, embora todos os medicamentos, exceto a atomoxetina, tenham afetado a qualidade do sono — reduzindo REM total, fase não REM, tempo de sono de ondas lentas e maior latência de início do sono.

**Conclusões:**

Os fármacos utilizados no tratamento do TDAH costumam cursar com repercussões negativas sobre a qualidade do sono de crianças, e o fármaco atomoxetina demonstrou menores efeitos sobre essa variável.

## INTRODUCTION

Attention deficit hyperactivity disorder (ADHD) is part of the neurodevelopmental disorders; its manifestations usually begin in childhood and are characterized by inattention, hyperactivity, and impulsivity, which last for a minimum period of 6 months and cause negative impact on the functioning and development of the individual, including their professional, academic, and social lives.^1^

This disorder has a prevalence of 2–7% in the community, being higher in boys than in girls, with a ratio of 3:1, but these data are doubtful, since there is difficulty in diagnosing ADHD, especially in girls, thus generating underdiagnosis in most countries.^2^ In addition, ADHD usually persists into adulthood and is a risk factor for other mental health disorders, with 75% of those with this disease having other disorders, thus affecting the prognosis of these patients.^3^

Some consequences of this disease are evidenced through somatic symptoms, especially in sleep disorders. A study carried out in the United States showed that children with ADHD have more resistance at bedtime, have difficulty initiating sleep, wake up more often during the night, and are resistant to waking up in the morning.^4^ Still in this perspective, a Spanish study objectively compares, through polysomnography, the sleep of children with and without ADHD, noting differences, especially in stage 1 of sleep, in which children with hyperactivity spent more time in this stage, representing the sleep changes that occur in this disorder.^5^ In addition, it is important to emphasize that sleep disorders are commonly associated with some comorbidities, such as arterial hypertension, obstructive sleep apnea syndrome, diabetes mellitus, increased insulin resistance, obesity, and dyslipidemia.^6^

Regarding the treatment of ADHD, behavioral therapy is the first line of treatment for preschool-aged children, as it reduces symptoms and improves the parent-child relationship, without the need to initially use pharmacotherapy. However, if symptoms persist or worsen, the use of psychostimulants is recommended as a second-line treatment, as these stimulant drugs work by improving attention span, hyperactivity, and impulsivity, despite having important adverse effects, such as sleep disturbances in patients.^7^

Thus, the present scientific study aimed to present an analysis of the effect of pharmacological treatment on the sleep pattern of children with ADHD.

## METHOD

A high-sensitivity electronic search was performed, as recommended by the Cochrane Handbook, and was peer reviewed according to the PRESS Guide. The search was carried out in May 2021 in the following databases: Cochrane Library, MEDLINE via PubMed, LILACS via Portal Regional da Saúde (BVS), Embase, Scopus, CINAHL, and Web of Science.

This systematic review complies with the recommendations and criteria described in the preferred reporting items for systematic reviews and meta-analyses (PRISMA)^8^ and Cochrane Handbook.^9^

Randomized clinical trials that were complete and available in full were included; they evaluated the relationship between pharmacological treatment in children with ADHD and their sleep pattern. They were conducted in a time frame between 2010 and 2020, and there was no language restriction.

The exploratory research question and definition of the study selection criteria were established according to the PICO mnemonic: “What is the effect of pharmacological treatment in children with ADHD in the sleep pattern?” The studies were first separated as to their typology and later analyzed as to their outcomes. All works that did not meet these inclusion criteria were excluded.

Platforms that were in accordance with the research database were used, so a refined search of electronic data was carried out, in order to identify the articles most consistent with the PICO strategy of the present study, which is described below:

P: “Attention Deficit Disorders with Hyperactivity” [MeSH] AND Child [MeSH]
I: “Drug Therapy” OR Methylphenidate [MeSH] OR “Lisdexamfetamine Dimesylate” [MeSH] OR “Atomoxetine Hydrochloride” [MeSH] OR Imipramine [MeSH] OR Clonidine [MeSH]
C: No intervention/placebo/sham
O: Sleep” [MeSH] OR sleep pattern [Tex Word]

After searching the databases, all studies were exported to a Mendeley^®^ file, and the duplicates were removed. Then, the file without duplicates was exported to the Rayyan QCRI tool, where two independent researchers (AD) and (RC) selected the articles by title and abstract. Then, the studies were analyzed in full according to the eligibility criteria. Results were summarized using the PRISMA flow diagram. In cases where there were conflicts, an independent researcher (NR) resolved the disagreements.

The final references were exported to a Google Sheets file, where the authors and the three independent authors (NR, AD, and RC) used a form to extract the characteristics of the studies. The data that were extracted are study data (authors, journal name, country, and place of study and year of publication), sociodemographic and clinical information, methodological information (design, total sample size, and interventions), and also data on the pattern of the study participants’ sleep.

Two review authors (AD and RC) assessed the quality of the studies blindly and independently, with disagreements being resolved through discussions between the raters. All included articles had their methodological quality assessed, but without using it as an exclusion criterion.

The Revised Cochrane Risk-of-bias Tool for Randomized Trials was used, a Cochrane tool recommended for analyzing the risk of bias in randomized clinical trials. This instrument has five domains of risk for bias, based on evidence and theoretical considerations:

1. Randomization process,
2. Deviations from intended interventions,
3. Lack of outcome data,
4. Result measurement, and
5. Selection of the reported outcome.

An Excel tool provided by Cochrane was used to organize the individual judgment of each author on the risks of bias and to generate the graph and summary of the risk of bias.

## RESULTS

**Figure 1.**
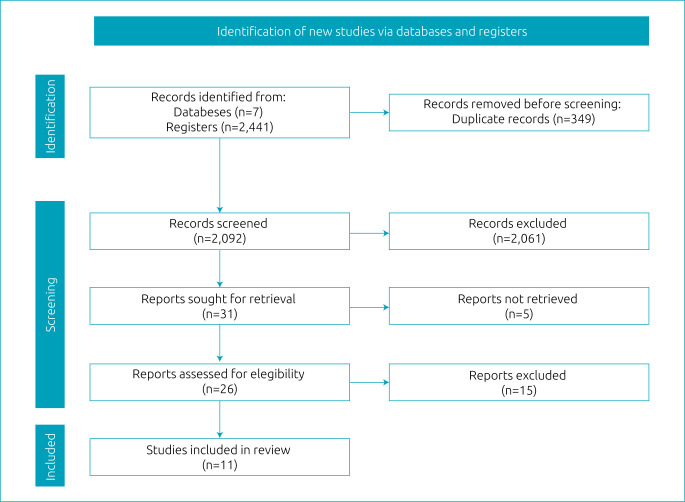
PRISMA flow diagram.

In total, 2,441 studies were located through searches carried out in 7 databases, with 293 texts found in MEDLINE via PubMed, 1,213 from the Cochrane Library, 118 from EMBASE, 2 from Web of Science, 8 from CINAHL, 18 from SCOPUS, and 789 from the VHL. After excluding 349 duplicates, 2,092 titles and abstracts were screened, resulting in 31 studies for full-text screening, of which 11 were included in the review, based on eligibility criteria and as described in the PRISMA flow diagram (Figure 1).

This review has only examined randomized clinical trials, totaling a population of 2,010 children. Regarding the geographic location of the included studies, we evaluated references from America (8), 6 from the United States of America and 2 from Canada; Europe (2), from Sweden and Holland. One study did not provide location information. All of them were published in English, between 2011 and 2020. Additional information about the studies is provided in Table 1.^10-20^

**Table 1.** Characteristics of studies.

Authors and year of publication	Total population	Age (years)	Intervention drug	Intervention duration	Instrument
Hollway et al.^10^, 2017	128	5–14	Atomoxetine	10 weeks	Children’s Sleep Habits Questionnaire
Rugino^11^, 2014	29	6–12	Guanfacine extended release (GXR)	5 weeks	Polysomnography and Children’s Sleep Habits Questionnaire
Ricketts et al.^12^, 2018	576	7–9	Methylphenidate	14 months	Achenbach Child Behavior Checklist 6-18
Goldman et al.^18^, 2018	342	6–12	Dasotraline	6 weeks	Children’s Sleep Habits Questionnaire
Owens et al.^13^, 2016	256	Study 1: 6–12 Study 2: 6–18	Methylphenidate HCl extended-release capsules	16 weeks	Children’s or Adolescent Sleep Habits Questionnaire
Becker et al.^17^, 2016	163	7–11	Methylphenidate	4 weeks	Pittsburgh Side Effects Rating Scale
Stein et al.^16^, 2015	230	Uninformed	Methylphenidate and atomoxetine	3–7 weeks	A sleep assessment scale validated and completed by parents
Lyon et al.^19^, 2011	93	8–12	L-Theanine chewable tablets	6 weeks	Pediatric Sleep Questionnaire and actigraphy
Solleveld et al.^14^, 2020	50	10–12	Immediate-release methylphenidate	16 weeks	Actigraphy, Holland Sleep Disorder Questionnaire; Epworth Sleepiness Scale, and Evaluation List Insomnia Therapy
Ashkenasi^15^, 2011	26	6–12	Transdermal methylphenidate	4 weeks	Daily updated sleep diary
Giblin and Strobel^20^, 2011	24	6–12	LDX administration	3 weeks	Polysomnography, actigraphy, and CSHQ

For intervention, the most commonly used drug was methylphenidate (MPH) (n=1,394), followed by atomoxetine (ATX) (n=358). Other drugs used were guanfaxine (n=29), dasotraline (n=342), L-theanine (n=93), and lisdexamfetamine (LDX) (n=24).

For the measurement of intervention-based studies, 6 of the 12 included studies used the Children’s Sleep Habits Questionnaire (CSHQ); other questionnaires used were the Pittsburgh Side Effects Rating Scale (PSERS), M-TuCASA, Holland Sleep Disorder Questionnaire (HSDQ), Epworth Sleepiness Scale (ESS), Evaluation List Insomnia Therapy (ELIT)*,* and Pediatric Sleep Questionnaire (PSQ). Five studies chose to use an objective measurement instrument, two studies of which used actigraphy, an exam that uses a device on the wrist for 1*–*4 weeks to assess the pattern of sleep and wakefulness; one study used polysomnography, an exam that assesses respiratory, muscular*,* and brain activity during sleep; and two studies used both methods.

**Figure 2.**
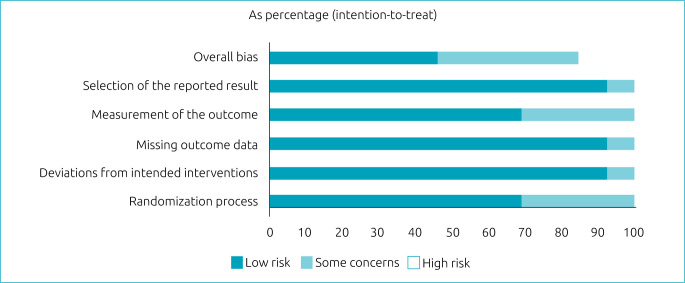
Risk of bias graph: judgments of the review authors on each risk of bias item in the included studies (presented as percentage).

**Figure 3.**
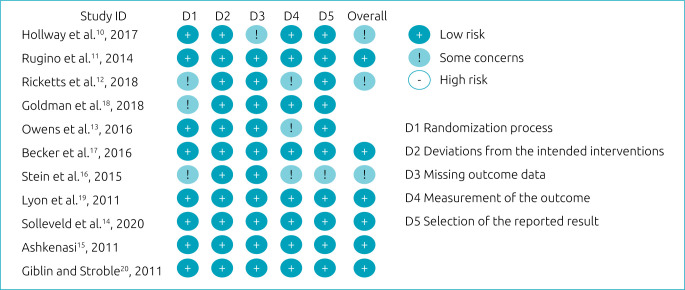
Summary of risk of bias: judgments by review authors on each risk of bias item in included studies.

Figure 2 shows the characteristics of the included studies and their risk of bias, and Figure 3 ^10-20^ shows the risk of bias summary: review authors’ judgments on each risk of bias item in the included studies. For randomized clinical trials, the following five risks of bias domains were considered:

1. Randomization process: low-risk articles were those that randomized the allocation sequence and masked it until participants were enrolled and allocated to the study. Furthermore, the presence of differences at baseline between the intervention groups was considered for classification between moderate and high risk.
2. Deviations from planned interventions: low-risk, double-blind articles were considered, in which the participants, as well as the caregivers or persons responsible for delivering the intervention, were not aware of the assigned intervention.
3. Absence of outcome data: articles in which outcome data were available for all or nearly all participants were considered low risk. “Almost all” should be interpreted as a small number of data losses that do not interfere with estimating the effect of the intervention.
4. Analysis of the results: articles in which the results assessor is not aware of the assigned intervention were considered low risk. The validity of the scales used and differences in measurement or evaluation between the intervention groups were also considered.
5. Selection of reported results: low-risk articles were those in which data were produced according to a pre-specified analysis that was finalized before the results were blinded. In addition, for articles with more than one scale, the adequate report of each was also considered.

## DISCUSSION

In this systematic review, 11 randomized clinical trials were included. All of them aimed to identify effects on sleep from drugs used to treat ADHD in children. Among the drugs studied are ATX, guanfacine, MPH, dasotraline, L-theanine, and LDX.^21^

Hollway et al.^10^, from the division into four intervention groups (ATX and parent training, only ATX, parent training, placebo, and only placebo), found that the drug had a nonsignificant effect on the hours of sleep, being, according to the study, indicated for patients who present insomnia or who later develop insomnia with stimulant treatment. In addition, it should be mentioned that this element is correlated with nonlinear kinetics due to factors influencing metabolism and concentration, and, therefore, physicians should pay attention to dose titration.^22^

Rugino^11^ evidenced, through the extended administration of guanfacine, that the symptoms of ADHD improved significantly; however, it provoked important sleep alterations in the polysomnography. For example, it increased awake time after sleep onset and reduced total rapid eye movement (REM), non-REM, and slow-wave sleep time. In this regard, the use of this medication in children and adolescents for the treatment of ADHD symptoms has already been identified as safe and effective, presenting, like most adverse effects, mild to moderate phenomena.^23^

MPH, in turn, was approached in different ways in the included studies; for example, Ricketts et al.^12^ used this drug in combination with behavioral therapy and showed effectiveness in reducing reports of sleep problems caused by the drug. In contrast, Owens et al.^13^ chose to investigate the extended release with capsules, having found a negative effect on the sleep of the sample, especially impacting the onset of this. In the immediate release of the drug, evaluated in the work by Solleveld et al.,^14^ efficiency and sleep duration were observed 1 week after the end of the trial, and the authors also mention that the evaluation of sleep problems soon after the beginning of the treatment can be inappropriate. Ashkenasi^15^ administered MPH transdermally and showed that there was no influence on sleep latency or total sleep time, but there was a trend toward improved sleep quality with longer patch use times.

In addition, a comparison was made between MPH and ATX in the same study; MPH was associated with a longer sleep-onset latency compared to ATX and a slightly longer sleep duration on weekends.^16^ In the attempt at monotherapy by Becker et al.,^17^ there was a general association between increasing the dose and increased sleep problems, although a proportion of children with preexisting sleep difficulties no longer had sleep problems with the highest dose of MPH.

In short, MPH in the treatment of ADHD in children can contribute to interpersonal relationships, increase concentration, and decrease aggression, although side effects are found, including the repercussions on sleep mentioned by the studies presented above.^24^

The use of dasotraline evaluated by Goldman et al.^18^ culminated in the identification of insomnia as an adverse event more commonly in the group that received the drug compared to patients treated with placebo. In this regard, it has already been used in the clinical trial by Findling et al.,^25^ who also pointed to insomnia as one of the main adverse effects. Through chewable L-theanine tablets, boys with ADHD had fewer episodes of nocturnal activity and an increased percentage of time spent in restful sleep, although there was no significant difference between groups (intervention vs. placebo) for sleep latency or duration.^19^ It is known that theanine also had positive results in the adult population, specifically aimed at promoting mental health in individuals with stress-related and cognitive impairments.^26^

Finally, LDX, when administered to the sample, had effects with little impact on tests performed to assess sleep, such as polysomnography and actigraphy; thus, LDX does not seem to contribute to any sleep disorders. The sample used in this study was small, and the multifaceted nature of the findings indicated that the study’s conclusions needed to be interpreted with caution and that more studies are needed on the influence of LDX on sleep in larger samples of children with ADHD.^20^ Thus, these findings were convergent with those of Mattos,^27^ where its effectiveness was evidenced and adverse effects, when present, were considered mild to moderate.

In short, most studies indicate positive results in the management of ADHD symptoms with the drugs used; however, significant changes in the sleep of children with this disorder are also mentioned.

Although there are no significant results, ATX shows relevant results by not altering sleep-related variables. Therefore, it is a therapeutic choice that can be considered, especially for patients with a history of sleep problems due to other drugs previously used.

The importance of adequate sleep for individuals, including the pediatric population, is highlighted. In this regard, its potential to influence various health indicators is known, such as body composition, emotional regulation, growth, quality of life, insulin sensitivity, and blood pressure. One example is its influence on brain structures and functions, such as memory, attention, and blood supply to brain structures.^28^ Thus, it is necessary that sleep disorders in children with ADHD, whether due to the pharmacology of the treatment or the disorder itself, be managed seriously, since they can contribute to the existence of other organic dysfunctions. However, the articles do not refer to the presence of comorbidities due to sleep disorders in the individuals included in their studies, possibly because they were children; such variables were not present in a significant number.

This systematic review suggests that the drugs used in the treatment of ADHD tend to have negative repercussions on the sleep quality of this group, and the drug ATX, in preliminary results, showed lesser effects on this variable. In addition, considering the importance of adequate sleep in children and adolescents, there is a need for significant changes to undergo interventions to prevent impacts on the health and quality of life of individuals who already have a condition (ADHD) and need specific care and treatment. Finally, more studies focused on the effects of ADHD pharmacology on sleep or other variables in children are needed to better understand and manage this clinical condition and, consequently, improve the quality of life of these patients.

## Data Availability

The database that originated the article is available with the corresponding author.
